# Duplex Real-Time PCR Assays for the Simultaneous Detection and Quantification of *Botryosphaeriaceae* Species Causing Canker Diseases in Woody Crops

**DOI:** 10.3390/plants12112205

**Published:** 2023-06-02

**Authors:** Laura Romero-Cuadrado, Carlos José López-Herrera, Ana Aguado, Nieves Capote

**Affiliations:** 1 Andalusian Institute of Agricultural and Fisheries Research and Training (IFAPA), Center Las TorresAlcalá del Río, 41200 Seville, Spain; laura.romero.cuadrado@juntadeandalucia.es (L.R.-C.); ana.aguado@juntadeandalucia.es (A.A.); 2 Instituto de Agricultura Sostenible, CSIC, C/ Alameda del Obispo s/n, 14004 Córdoba, Spain; lherrera@ias.csic.es

**Keywords:** multiplex qPCR, almond, plant crude extracts, latent infection, *Neofusicoccum parvum*, *Botryosphaeria dothidea*

## Abstract

Woody canker diseases caused by fungi of the *Botryosphaeriaceae* family are producing increasing losses in many economically important woody crops, including almond. To develop a molecular tool for the detection and quantification of the most aggressive and threatening species is of main importance. This will help to prevent the introduction of these pathogens in new orchards and to conveniently apply the appropriate control measures. Three reliable, sensitive and specific duplex qPCR assays using TaqMan probes have been designed for the detection and quantification of (a) *Neofusicoccum parvum* and the *Neofusicoccum* genus, (b) *N. parvum* and the *Botryosphaeriaceae* family and (c) *Botryosphaeria dothidea* and the *Botryosphaeriaceae* family. The multiplex qPCR protocols have been validated on artificially and naturally infected plants. Direct systems to process plant materials, without DNA purification, allowed high-throughput detection of *Botryosphaeriaceae* targets even in asymptomatic tissues. These results validate the qPCR using the direct sample preparation method as a valuable tool for Botryosphaeria dieback diagnosis allowing a large-scale analysis and the preventive detection of latent infection.

## 1. Introduction

Almond (*Prunus dulcis* (Mill.) D.A. Webb) is one the most important nut crops in the world. California (USA) is the leader in almond production followed by Spain and Australia (FAOSTAT, 2021; http://www.fao.org/faostat/en/#data (accessed on 10 March 2023). The implementation of new management techniques, such as high-density cultivation, prune intensification, drip irrigation and fertilization, mechanical harvest, use of more productive varieties and the cultivation in agronomically and environmentally more favorable cropping areas, has increased the fruit production in the last decades [1]. However, this new scenario, together with the current climate change situation, have favored the increase in almond diseases [2,3,4]. Among them, woody canker diseases, associated with fungi of the *Botryosphaeriaceae* family, affect a great number of agronomically important woody crops such as olive, grapevine, avocado, blueberry, stone fruit, citrus and nut crops, including almond [5,6,7,8,9,10,11,12]. These diseases affect the trunk and branches of young and mature almond trees causing cankers, extensive gumming, dieback, discoloration and necrosis of internal tissues and, especially in severe cases and young trees, plant death. Almond canker diseases have been described in California [13,14,15,16,17], Iran [18], Turkey [19], Morocco [20] and Spain [5,21,22,23], causing important economic losses [24].

Fungi of the *Botryosphaeriaceae* family are one of the most prevalent pathogens in almond orchards [13,16,21]. Many *Botryosphaeriaceae* species have been associated with almond cankers worldwide (*Botryosphaeria dothidea*, *Diplodia corticola*, *D. gallae*, *D. mutila*, *D. olivarum*, *D. seriata*, *Dothiorella iberica*, *Do. prunicola*, *Do. sarmentorum*, *Do. viticola*, *Lasiodiplodia theobromae*, *Macrophomina phaseolina*, *Neofusicoccum arbuti*, *N. australe*, *N. luteum*, *N. mediterraneum*, *N. nonquaesitum*, *N. parvum*, *N. vitifusiforme* and *Neoscytalidium dimidiatum*) [5,13,15,16,18,21,22,23,25] with *Neofusicoccum* being the prevailing genus [24]. Regarding other nut crops, *N. parvum* is also the dominant species in walnut orchards in Australia, California, China, Chile, Italy, Iran and Spain [18,26,27,28,29,30,31,32], and *N. mediterraneum* prevails in Spanish and Californian pistachio orchards [33,34]. The infection of the host plant occurs through natural wounds or those caused by pruning or injury or through other openings such as lenticels and stomata. These fungi can act as saprophytic, endophytic or latent pathogens and disease symptoms usually appear when the host is under stress conditions [35,36]. Therefore, the latent infections allow the “silent” introduction of the pathogens in new plantations and a late diagnosis of the disease, making control measures less efficient. Diagnosis of the disease by symptoms visualization in the field is not accurate because canker symptoms caused by other non-*Botryosphaeriaceae* species can produce similar symptoms and, in addition, co-infection of a single plant by different pathogenic species usually occurs [16]. Traditional methods for the isolation and identification of *Botryosphaeriaceae* species are based on culture of symptomatic plant tissues in the appropriate culture medium, followed by obtention of monoconidial or hyphal tip isolates and further characterization based on morphological characteristics (colony aspect or microscopic features). This process is laborious and time-consuming and not always accurate. In addition, many of the *Botryosphaeriaceae* species do not produce conidia in culture medium [37], making identification difficult.

For the preventive detection and an accurate diagnosis of fungal diseases, the use of molecular tools based on real-time PCR is one of the most promising strategies. PCR-based methods (conventional PCR and nested PCR) have been designed to detect species of *Botryosphaeriaceae* in almonds [38] and in other crops [39] or for the specific detection of *Botryosphaeriaceae* family [40]. In addition, qPCR-based methods have also been designed for the detection of some species or genera in the *Botryosphaeriaceae* family [41,42,43,44]. Most of the previously designed qPCR protocols used SYBR Green chemistry, which is cheaper and extensively used for the detection of high number of targets. However, the use of hydrolysis probes such as TaqMan probes has been demonstrated to be more advantageous due to their higher specificity (an additional specific primer, the TaqMan probe, is used, while SYBR Green can unspecifically join to dsDNA and/or generate by products), higher sensitivity and, in addition, the possibility to accurately quantify the fungal target [45].

Among the *Botryosphaeriaceae* family, *Neofusicoccum parvum* and *Botryosphaeria dothidea* have been reported as the most aggressive and most prevailing species in Spanish almond crop, respectively [21,23]. However, since the accurate identification of the causal agents is not easy and canker diseases can be caused by more than one pathogen, the objectives of this work were (i) to develop sensitive and reliable qPCR methods based on TaqMan probes for the simultaneous detection of (a) *N. parvum* and the *Neofusicoccum* genus, (b) *N. parvum* and the *Botryosphaeriaceae* family and (c) *B. dothidea* and the *Botryosphaeriaceae* family, in almond; (ii) to validate the designed protocols in artificially and naturally infected almonds by using two methods of sample preparation: extraction of DNA from infected tissues and direct preparation of plant crude extracts; and (iii) to apply the designed duplex qPCRs for the detection of the latent infection of *Botryosphaeriaceae* species in almond.

## 2. Results

### 2.1. Design of Primers and Probes

Primers and probes for the specific detection of *N. parvum* and *B. dothidea* were designed based on sequences from the translation elongation factor 1-alpha gene (*tef1*). Primers and probes for the multispecies detection of *Neofusicoccum* spp. and *Botryosphaeriaceae* family were designed based on sequences from the beta tubulin gene (*tub2*) (Table 1). For the duplex detection, TaqMan probes specific for *N. parvum* and *B. dothidea* were labelled with FAM fluorophore and TaqMan probes for the detection of *Neofusicocum* spp. and *Botryosphaeriaceae* family were marked with SUN fluorophore.

### 2.2. Analytical Specificity—Inclusivity and Exclusivity—And Limit of Detection

Simplex qPCR protocols for the specific detection of *N. parvum* and *B. dothidea* did not detect other *Neofusicoccum* or *Botryosphaeria* species used in the specificity tests. Furthermore, BLAST analyses showed that *N. parvum*-specific qPCR protocol would not detect *Neofusicoccum* species associated to trunk diseases of walnut (*N. luteum*, *N. mediterraneum*, *N. nonquaesitum*, *N. vitifusiforme*) and pistachio (*N. australe*, *N. hellenicum*, *N. mediterraneum*, *N. pistaciae N. vitifusiforme*) [24,46] nor *Neofusicoccum* species affecting other Mediterranean crops, such as *Citrus* spp. and olive [47]. However, primers and probe for the detection of *N. parvum* could detect some of the phylogenetically closest species in the *N. parvum* species complex, such as *N. ribis* and *N. kwambonambiense,* which affect Mediterranean woody crops (Table S2). The *B. dothidea* simplex qPCR protocol could also detect *B. corticis* and *B. fabicerciana* described in *Prunus* and other woody crops in Mediterranean areas but not reported in almond [48,49] (Table S2). On the contrary, primers and probes designed for the specific detection of the genus *Neofusicoccum* and the *Botryosphaeriaceae* family could detect, respectively, all the *Neofusicoccum* and *Botryosphaeriaceae* species described in almond and Mediterranean crops. No amplification was detected using DNA from non-target species or negative controls.

**Figure 1:**
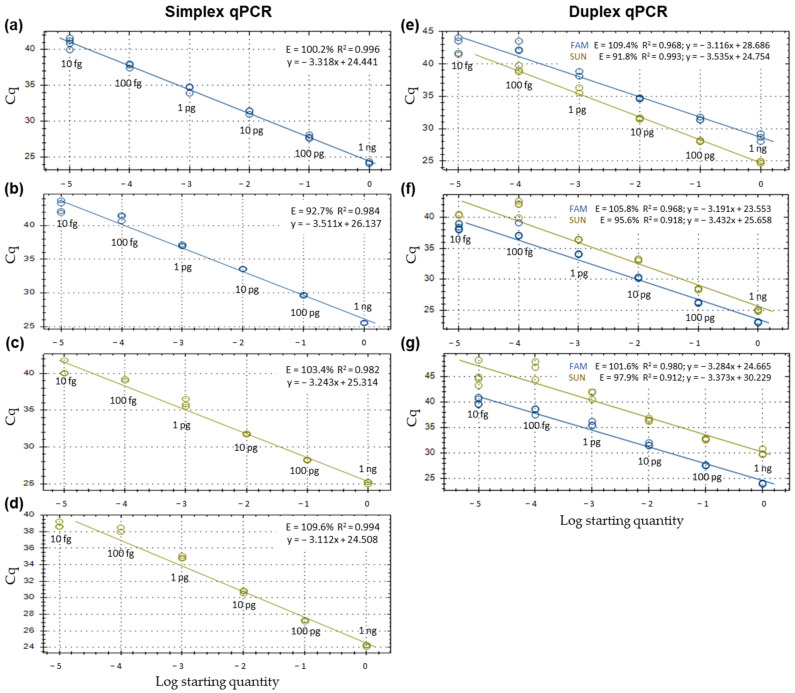
Standard curves in simplex and duplex qPCR for the quantitative detection of (**a**) *Neofusicoccum parvum*; (**b**) *Botryosphaeria dothidea*; (**c**) *Neofusicoccum* genus; (**d**) *Botryosphaeriaceae* family; (**e**) *N. parvum* and *Neofusicoccum* genus; (**f**) *N. parvum* and *Botryosphaeriaceae* family; and (**g**) *B. dothidea* and *Botryosphaeriaceae* family*. N. parvum* and *B. dothidea* were detected with FAM fluorophore*. Neofusicoccum* genus and *Botryosphaeriaceae* family were detected with SUN fluorophore. Ten-fold dilutions of genomic DNA from pure colonies of *N. parvum* and *B. dothidea* (1 ng to 10 fg) were amplified in three or six (10 fg point, limit of detection) replicates. Efficiency (E), coefficient of determination (R^2^) and regression equations of standard curves are shown for each qPCR reaction.

Simplex and duplex qPCR reactions showed efficiencies between 90 and 110% and R^2^ about 0.99 (Figure 1). Duplex qPCR reactions did not decrease the sensitivity of detection nor decrease R^2^ values compared with simplex qPCR reactions.

The limit of detection of *N. parvum*, *B. dothidea*, *Neofusicoccum* spp. and *Botryosphaeriaceae* family was 10 fg of genomic DNA from pure fungal colonies (see Material and Methods). Although it was possible to detect as low as 1 fg of genomic DNA in some of the replicates, the limit of detection was established from 10 fg of genomic DNA due to the reliability of the repetitions (Figure 1).

### 2.3. Detection of Botryosphaeria Species in Naturally and Artificially Infected Plants

Thirteen out of eighteen samples from naturally infected almonds cvs. ‘Soleta’, ‘Lauranne’, ‘Marcona’ and ‘Belona’ rendered positive results by using the developed qPCR methods (Table 2). *B. dothidea* and *N. parvum* were detected in 62.5% and 12.5% of the affected trees, respectively, by simplex and duplex qPCRs. Mixed infections of *Botryosphaeriaceae* species were not detected in any of the analyzed trees. Three ‘Soleta’ and two ‘Lauranne’ trees which presented canker symptoms (Sol 5, Sol 6, Lau 3 and Lau 4) rendered negative results for *Botryosphaeriaceae* species detection by qPCR, but *Cytospora* sp. was isolated from these trees by isolation and sequence identification. The simplex and the duplex qPCR for detection of *N. parvum* and *Neofusicoccum* genera yielded negative results when applied to almond trees naturally infected with *B. dothidea*. Similarly, simplex *B. dothidea*-specific qPCR yielded negative results when applied to trees naturally infected with *N. parvum.* Asymptomatic trees from ‘Soleta’ (Sol 10) and ‘Lauranne’ (Lau 6) cultivars were negative for simplex and duplex qPCR detection. The detection of *Botryosphaeriaceae* species by simplex or duplex qPCR was 100% consistent with the results obtained by isolation and sequencing (Table 2).

Artificially inoculated almond twigs and naturally infected samples were used to compare qPCR detection of *Botryosphaeriaceae* species by using DNA extracted with a commercial kit or directly prepared plant crude extracts. Detection of *N. parvum*, *B. dothidea*, *Neofusicoccum* genus and *Botryosphaeriaceae* family by direct qPCR using 1:10 dilution of plant crude extracts of artificially and naturally infected samples had 100% of coincident results with qPCR using DNA extractions, demonstrating high agreement between the two sample preparation techniques. The sensitivity of detection when using plant crude extracts was one order of magnitude lower than when using DNA extracted with a commercial kit. Additionally, plant crude extracts used without dilution (1:1; *v*:*v*) did not always give amplification signal. Therefore, a 1:10 (*v*:*v*) dilution of the plant crude extract is advisable for direct qPCR detection (Table 3).

### 2.4. Detection of Botryosphaeriaceae Species on Asymptomatic Plant Tissues

**Figure 2:**
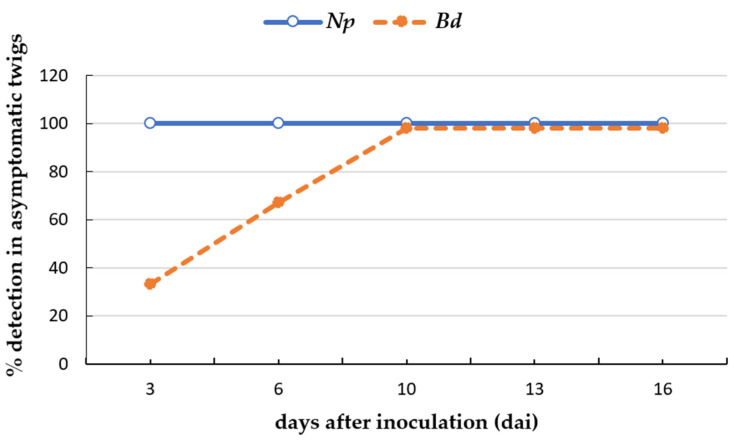
Incidence of latent infection of *Neofusicoccum parvum* (*Np*) and *Botryosphaeria dothidea* (*Bd*) in asymptomatic almond twigs artificially inoculated with isolates NpALM2 and BdALM2, respectively, measured at 3, 6, 10, 13 and 16 days after inoculation (dai).

The qPCR protocols developed using dilutions of plant crude extracts as sample could detect *N. parvum* and *B. dothidea* in artificially inoculated asymptomatic plant tissues. The first necrotic lesions appeared on some of the inoculated twigs 10 days after inoculation (dai) for *N. parvum* and *B. dothidea* but only the asymptomatic twigs were selected for the qPCR analysis. *N. parvum* was detected from 3 dai to 16 dai in all asymptomatic twigs assayed. *B. dothidea* was detected in 33% of the asymptomatic twigs analyzed at 3 dai and in 100% of the asymptomatic twigs from 10 to 16 dai (Figure 2). The amount of fungal DNA quantified in the asymptomatic twigs ranged from 11.6 fg at 3 dai to 1.09 ng at 16 dai for *N. parvum* and from 18.2 fg at 3 dai to 1.1 pg at 16 dai for *B. dothidea*. Both fungi were respectively detected from 10 dai in symptomatic twigs.

## 3. Discussion

Wood canker diseases are a major threat to global nut crop productivity. Among them, canker diseases caused by *Botryosphaeriaceae* fungi have not been given the importance they deserve and the role of these fungi in wood diseases has been overlooked. The fact that *Botryosphaeriaceae* species have a wide host range and produce latent infections has led to their “silent” introduction into crop fields through adjacent infected crops and infected but asymptomatic nursery plant material, to later disperse through rain, wind, insects or pruning and harvest tools. In fact, the number of reports associated with trunk diseases caused by *Botryosphaeriaceae* in numerous woody crops worldwide has significantly increased in the past years [50,51]. The preventive detection of the causal agents is one of the most important and effective strategies for the control of fungal diseases. For that reason, the development of an accurate, sensitive and rapid diagnostic method is of great importance. In this work, molecular tools based on TaqMan-qPCR protocols have been developed for the specific detection and quantification of the two most important *Botryosphaeriaceae* species of the almond crop: *N. parvum* and *B. dothidea*. However, as these two species are not the only ones that infect almond, and more than one species is usually detected in a producing orchard and in a single infected plant, duplex qPCR have been designed to detect the genus *Neofusicoccum* and the family *Botryosphaeriaceae,* thus giving information about the presence of other unknown *Botryosphaeriacae* species.

One of the main advantages of the designed qPCR protocols is their high sensitivity of detection compared with other PCR-based methods previously reported. Conventional and nested PCR performed in multiplex reached a detection limit of 1 pg and 0.1 pg of *N. parvum* gDNA, respectively [38,39,40] and 100 pg of *B. dothidea* gDNA [38]. These methods are time consuming and require visualization of the results in agarose gels. This entails the risk of cross contamination and, therefore, the appearance of false positives. Previously reported qPCR methods for detection of canker-causing pathogens generally used SYBR Green chemistry [42,44]. Those using hydrolysis probes were more sensitive and were able to detect as low as 200 fg of *N. parvum* gDNA [43]. Our methods have improved the sensitivity of detection, even allowing the detection of *Botryosphaeriaceae* species in asymptomatic tissues. In addition, the implementation of a duplex qPCR for the simultaneous detection of several targets at the same time and the use of a direct sample preparation method allow the analysis of a large number of samples, saving costs. One of the main constraints of the designed qPCR protocols is the impossibility to distinguish between *N. parvum* and some of the phylogenetically closest species within the *N. parvum* species complex such as *N. ribis* and *N. kwambonambiense* or between *B. dothidea* and *B. corticis* and *B. fabicerciana.* These species are not described in almond but affect some Mediterranean woody crops such as blueberry, grapevine, citrus and avocado [25,49,52,53]. If a precise identification is required, isolation of the pathogen and subsequent morphological identification or sequencing of another genome locus would be necessary.

Several studies have evidenced the endophytic phase of *Botryosphaeriaceae* fungi in nursery plant material [54,55,56,57]. In this sense, the high sensitivity of the developed qPCR tools allows for the early detection of these pathogens even before the appearance of disease symptoms, providing evidence of the latent infection of these canker-causing pathogens in woody plant tissues. Even more, using the direct sample preparation method described in this study allows the friendly and more economic analysis of a large number of samples. Detection of the target fungi failed when using non diluted plant crude extracts probably due to the presence in the reaction of high amounts of PCR inhibitors of plant origin. Using bovine serum albumin (BSA, 0.1 mg/mL) in the qPCR reaction helped to detect more true positives, but not all. Therefore, the possibility of detecting false negatives makes it advisable to use 1:10 dilutions and BSA which showed high agreement with the DNA extraction method. Therefore, these tools could be applied for the preventive detection of *Botryosphaeriaceae* fungi in recently planted young trees and in nursery plant material, even being certified as free of the aforementioned pathogens, ensuring the sale of healthy plants to the farmer and, at the same time, avoiding the introduction and dispersion of these pathogens in production fields.

The qPCR protocols described in this work are useful not only for the accurate pathogen identification and disease diagnostics in almond, but also for their application in epidemiological studies to determine the source of inoculum (air, irrigation water, neighboring crops) and quantify the seasonal variation of the inoculum level and the patterns of dispersion of *Botryosphaeriaceae* spores in almond orchards as previously described [43]. Additionally, this knowledge will permit to establish the correct schedules for the application of control measures, thus avoiding economic losses and damage to the environment. Validation of these qPCR protocols for detection of *Botryosphaeriaceae* groups in environmental samples should be addressed. In addition, these molecular tools could be applied to evaluate the efficacy of fungicide or biological control agents treatments, by quantifying the time-course concentration of fungal inoculum before and after treatments, as previously reported in other pathosystems [58].

In conclusion, this study provides an accurate, sensitive and easy-to-use molecular tool for the detection of the most important botryosphaeriaceous canker-causing pathogens, *N. parvum* and *B. dothidea*, along with any other species from the *Neofusicoccum* genus or *Botryosphaeriaceae* family in almond. These qPCR protocols could be potentially also applied to other woody crops affected by these diseases, after appropriate validation, i.e., the verification that the variation in the specific matrix does not affect the test performance.

## 4. Materials and Methods

### 4.1. Surveys of Almond Orchards and Fungal Isolation

Three commercial almond orchards affected by almond trunk diseases located in Alcalá del Río, La Rinconada and Jerez de la Frontera locations (southwestern Spain) were surveyed in 2021 and 2022. Symptomatic plant material exhibiting trunk cankers, gummosis and internal necrosis were taken to be analyzed in the laboratory. Samples were used for both the isolation of *Botryosphaeriaceae* fungi in culture medium and for the detection of *Botryosphaeriaceae* species using qPCR protocols. For the isolation of fungal isolates, plant tissues were surface-disinfected in 1.5% sodium hypochlorite solution for 2 min, rinsed twice in sterile distilled water, and left to air dry in a laminar flow cabinet. Small pieces were plated on potato dextrose agar supplemented with 0.5 g/L of streptomycin sulphate (Sigma-Aldrich, St. Louis, MO, USA) (PDAS). Petri dishes were incubated at 25 °C in darkness for 7–10 d and any *Botryosphaeriaceae*-like colony was individually transferred to PDA plates. All isolates were hyphal-tipped and maintained at −80 °C in cryovials containing 20% glycerol and at 4 °C in vials containing sterile distilled water, in the fungal collection of IFAPA Centro Las Torres (Seville, Spain).

The rest of fungal isolates used in this study were *Botryosphaeriaceae* species isolated from other woody crops, such as avocado and blueberry affected by trunk diseases [8,10], fungal genera phylogenetically close to the target species, and non-*Botryosphaeriaceae* pathogenic or endophytic species from almond (Table 4).

### 4.2. Fungal DNA Extraction and Identification by Sequencing

Fungal genomic DNA was extracted from pure fungal cultures using 0.1 mg of mycelium scraped from PDA plates incubated at 25 °C in darkness for 5–7 d using the HigherPurity Plant DNA Purification Kit (Canvax Biotech, S.L., Córdoba, Spain) and following the instructions of the manufacturer. DNA concentration was quantified using a NanoDrop 2000 Spectrophotometer (Thermo Scientific, Wilmington, DE, USA).

For identification, sequencing of the internal transcribed spacer (ITS) nuclear rDNA using ITS1 and ITS4 primers [59] and a portion of the *tef1* gene using EF446f and EF1035r primers [60] was performed. The sequences generated were deposited in the GenBank (Table S1) and compared with available sequences by BLAST analysis.

### 4.3. Design of qPCR Protocols

Firstly, simplex qPCR protocols were designed for the single detection of *N. parvum*, *B. dothidea*, *Neofusicoccum* spp. and the *Botryosphaeriaceae* family, respectively. Then, conditions for duplex qPCR for the simultaneous detection of *N. parvum*/*Neofusicoccum* spp., *N. parvum*/*Botryosphaeriaceae* family and *B. dothidea*/*Botryosphaeriaceae* family were optimized:

#### 4.3.1. Design of Specific Primers and Probes

Sequences of the ITS region, *tef1* gene, and *tub2* gene from *Botryosphaeriaceae* species were retrieved form the GenBank and aligned using Mega 7.0 software [61]. Nucleotide sequences among the three loci that showed the highest specific consensus for each fungal target and not for the remaining species were selected and used to design forward/reverse primers and TaqMan probes using the software from Integrated DNA Technologies, Inc. (IDT) under its default settings. A BLASTn query against the NCBI GenBank database was used to ensure the specificity of the primers and probes in silico (Table S2). TaqMan probes were labeled with 6-carboxy-fluorescein, FAM (for *N. parvum* and *B. dothidea*) and SUN (for *Neofusicoccum* spp. and *Botryosphaeriaceae* family) reporter dyes at the 5′-end. TaqMan probes also harbored an internal ZEN quencher and an Iowa Black FQ quencher (IBFQ) at the 3′-end.

#### 4.3.2. Optimization of qPCR Conditions

Combination of several primers and probes concentrations were tested to obtain the best efficiency reaction and higher sensitivity. Final concentrations of 300, 600 and 900 nM of primers and 100, 150, 250 nM of probes were assayed. Each DNA sample was run in simplex and duplex reactions to compare reaction efficiency and sensitivity. qPCR assays were performed in 96-well plates using a CFX Connect thermocycler (Bio-Rad, Hercules, CA, USA) in a 20 μL reaction volume. Reaction cocktails contained sample template (5 μL of extracted DNA from a pure fungal colony or from infected plant tissue or 1:10 (*v*:*v*) dilution of plant crude extracts), iTaq Universal Probes Supermix (1×) (Bio-Rad, Hercules, CA, USA), forward and reverse primers for *N. parvum* and *B. dothidea* (600 nM) or for *Neofusicoccum* spp. and *Botryosphaeriaceae* family (300 nM), TaqMan probes for *N. parvum* and *B. dothidea* (250 nM) or for *Neofusicoccum* spp. (100 nM) and *Botryosphaeriaceae* family (200 nM). Amplifications were performed at 95 °C for 5 min, then 45 cycles of 5 s at 95 °C, followed by 40 s at 60 °C. In each run, sterile distilled water was used instead of DNA as a no-template control. The data were analyzed using the CFX Maestro software 2.3 (Bio-Rad, Hercules, CA, USA). Once simplex qPCR reactions were optimized, duplex qPCRs were performed for the simultaneous detection of (a) *N. parvum* and *Neofusicoccum* genus, (b) *N. parvum* and *Botryosphaeriaceae* family and (c) *B. dothidea* and *Botryosphaeriaceae* family. Conditions for duplex qPCR were the same as for simplex qPCR.

### 4.4. Analytical Specificity and Analytical Sensitivity of the qPCR Reactions

The analytical specificity, inclusivity and exclusivity of each individual and duplex qPCR were tested on DNA from 81 isolates including species of the *Botryosphaeriaceae* family and other fungal species pathogenic and non-pathogenic to almond (Table 4). In addition, BLAST analysis was also performed using forward and reverse primers and probe sequences of each *Botryosphaeriaceae* target separately on the corresponding sequences of all described *Botryosphaeriaceae* species affecting almond and other Mediterranean crops (Table S2).

The analytical sensitivity of the qPCR assays was evaluated using eight ten-fold decreasing concentrations of DNA obtained from pure colonies of *N. parvum* NpALM2 isolate and *B. dothidea* BdALM2 isolate (0.2 ng/μL to 0.2 fg/μL) diluted in a background of DNA (10 ng/μL) extracted from the subcortical tissue of a healthy almond plant. An amount of 5 μL of each dilution was used as template in the qPCR reactions (1 ng to 1 fg of total genomic DNA/qPCR reaction). For each dilution point, we amplified three replicates (from 1 ng to 100 fg/qPCR reaction) and six replicates (for 10 and 1 fg/qPCR reaction) and the values obtained for each dilution series were used to generate standard curves for quantification of the respective fungal targets in simplex and duplex qPCR reactions. Each qPCR assay was repeated at least three times. Linear regression between the quantification cycle (Cq) and the log value of DNA concentration was performed to obtain the corresponding quantification values.

### 4.5. Plant DNA Extraction and Direct Sample Preparation Method

Samples from almond trees (small pieces of inoculated twigs and trunk subcortical tissue) were divided into two halves: one half was grounded in a mortar in the presence of liquid nitrogen and 0.1 mg of the homogenized tissue were used for DNA isolation following the instructions of the DNeasy Plant Pro kit (Qiagen, Hilden, Germany). DNA concentration was measured using a NanoDrop 2000 Spectrophotometer (Thermo Scientific, Wilmington, DE, USA) and stored at −20 °C for further analysis; the other half was introduced into plastic bags containing a soft net (Bioreba, Reinach, Switzerland) and grounded with the help of a manual homogenizer (or a hammer in the case of trunk subcortical tissues), in the presence of 1:20 (*w*:*v*) PBS buffer, pH 7.2 supplemented with 2% (*w*:*v*) polyvinyl pyrrolidone and 0.2% (*w*:*v*) sodium diethyl dithiocarbamate to obtain a plant crude extract. An amount of 5 μL of kit-extracted DNA and 5 μL of plant crude extracts were used in the qPCR reactions. Serial dilutions (1:1 to 1:10^7^ *v*:*v*) of extracted DNA and plant crude extracts from the same tissue were used for qPCR approaches to compare the sensitivities of the two methods.

### 4.6. Validation of the Assays in Naturally and Artificially Infected Tissues

For validation of the qPCR protocols in naturally infected plants, subcortical tissue from the trunk of symptomatic and asymptomatic (negative control) almond trees were collected, surface sterilized in 1.5% sodium hypochlorite for 2 min, rinsed twice in sterile distilled water and air-dried. Small pieces (0.5 cm) were taken for both, the reisolation of inoculated fungi in PDA plates and qPCR detection. The pieces selected for fungal reisolation were placed on PDA plates and incubated at 25 °C for 7–10 days in darkness. The pieces selected for qPCR detection were divided into two halves, one for DNA extraction and the other for the preparation of plant crude extracts (see Section 4.6). For all samples, qPCR reactions were performed for the simplex detection of *N. parvum* and *B. dothidea* and the duplex detection of *N. parvum* + *Neofusicoccum* spp., *N. parvum* + *Botryosphaeriaceae* family and *B. dothidea* + *Botryosphaeriaceae* family.

For validation of the qPCR assays in artificially infected almonds, one-year-old twigs from the ‘Guara’ cultivar were inoculated with representative isolates of *N. parvum* (isolate NpALM2) and *B. dothidea* (isolate BdALM2). For this, the almond twigs were cut to 12 cm in length, surface sterilized by spraying with 75% ethanol to run off and air dried in a laminar flow cabinet under sterile conditions. Two wounds per twig were made with a 5-millimeter-diameter cork borer. Five-millimeter mycelium plugs from colonies actively growing on PDA were placed in the wounds and sealed with parafilm. Inoculated twigs were individually inserted in glass tubes containing 2 mL of sterile water. Tubes were capped and incubated in a growth chamber at 25 °C. Eight twigs per isolate were inoculated and twigs with non-colonized PDA plugs were used as negative controls. Two weeks after inoculation, the twigs were observed and small pieces from the margin of necrotic lesions were taken for both the reisolation of inoculated fungi in PDA plates and DNA extraction for qPCR detection, as explained above.

To confirm the specificity of the qPCR protocols, plant crude extracts and gDNA extracted form plant material artificially and naturally infected with *N. parvum* or *B. dothidea* were used as template in all simplex and duplex qPCR reactions.

### 4.7. Detection of Botryosphaeriaceae Fungi in Asymptomatic Tissues

To ascertain whether the qPCR protocols designed were able to detect *Botryosphaeriaceae* fungi in asymptomatic plant tissues, *N. parvum* NpALM2 and *B. dothidea* BdALM2 isolates were artificially inoculated on respective detached twigs of almond cv. Guara as explained above. Thirty twigs were inoculated per pathogen isolate. Successive sampling was performed at 3, 6, 10, 13 and 16 dai. At each sampling time, 5 twigs without symptoms were selected. Samples consisted of pieces (1 × 0.5 mm) of subcortical tissue without epidermis taken from the left and right of the inoculation site, 2 mm apart from the inoculation point. The sampled tissue fragments were processed to extract DNA and prepare plant crude extracts, as described above. Duplex qPCR protocols were applied to detect *N. parvum* and *B. dothidea*, respectively, and the percentage of detection in asymptomatic twigs was calculated.

## Figures and Tables

**Table 1:** Primers and TaqMan probes designed for the specific detection of *Botryosphaeriaceae* species by simplex and duplex qPCR.

Target Organism	Oligo Name	Oligo Type	Sequence 5’–3’	Target Gene ^1^
*Botryosphaeria dothidea*	Bd-F1	Forward primer	CGCCGAATTTGCCTTATCA	*tef1*
	Bd-R1	Reverse primer	TTAGCATATGGTCGCATAGAC	
	Bd-P	Probe	FAM-TCACCAACG/ZEN/CTTCCAGCCACTCA-IABkFQ	
*Neofusicoccum parvum*	Np-F1	Forward primer	GAAGTTCGAGAAGGTAAGAAAGT	*tef1*
	Np-R1	Reverse primer	TGAGTGCGGGAACCC	
	Np-P	Probe	FAM-CTGCACGCG/ZEN/CTGGGTGCCAG-IABkFQ	
*Neofusicoccum* spp.	Nspp-F	Forward primer	GGCCTGGACGGCTCT	*tub2*
	Nspp-R1	Reverse primer	AGTGAGAGAGTACCTCGTTGAAG	
	Nspp-P	Probe	SUN-GCGCGAATG/ZEN/GCAATGGCTGACC-IABkFQ	
*Botryosphaeriaceae* family	Bot-F1	Forward primer	GTATGGCAATCTTCTGAACG	*tub2*
	Bot-R2	Reverse primer	GAARAGCTGGCCRAAGG	
	Bot-P	Probe	SUN-TCGAGCCCG/ZEN/GCACSATGGAT-IABkFQ	

^1^ *tef1*: translation elongation factor 1-alpha; *tub2*: beta-tubulin 2. IABkFQ: Iowa Black^®^FQ.

**Table 2:** Concordance between molecular and traditional methods (simplex and duplex qPCR using extracted DNA as sample and isolation methods) for the detection of *Botryosphaeriaceae* species in subcortical tissue of naturally infected almond trees.

Almond Tree Samples ^1^	Simplex qPCR ^2^		Duplex qPCR ^2^			Isolation and Sequencing
	*Np*	*Bd*	*Np + N*spp.	*Np + Bot* Family	*Bd + Bot* Family	
Mar 1	−	+	−	+	+	*B. dothidea*
Sol 2	−	+	−	+	+	*B. dothidea*
Sol 3	−	+	−	+	+	*B. dothidea*
Sol 5	−	−	−	−	−	*Cytospora* sp.
Sol 6	−	−	−	−	−	*Cytospora* sp.
Sol 7	+	−	+	+	+	*N. parvum*/*Cytospora* sp.
Sol 9	+	−	+	+	+	*N. parvum*
Sol 11	−	+	−	+	+	*B. dothidea*
Lau 2	−	+	−	+	+	*B. dothidea*
Lau 3	−	−	−	−	−	*Cytospora* sp.
Lau 4	−	−	−	−	−	*Cytospora* sp.
Lau 7	−	+	−	+	+	*B. dothidea*
Bel 3	−	+	−	+	+	*B. dothidea*
Bel 4	−	+	−	+	+	*B. dothidea*
Bel 5	−	+	−	+	+	*B. dothidea*
Bel 6	−	+	−	+	+	*B. dothidea*
Sol 10 *	−	−	−	−	−	NI
Lau 6 *	−	−	−	−	−	NI

^1^ Almond trees of cultivars ‘Marcona’ (Mar), ‘Soleta’ (Sol), ‘Lauranne’ (Lau) and ‘Belona’ (Bel). Asterisks indicate asymptomatic almond trees; ^2^ + Positive qPCR amplification; − Negative qPCR amplification. NI: no canker-causing fungi isolation. *Np*: *Neofusicoccum parvum*; *Bd*: *Botryosphaeria dothidea; N*spp.: *Neofusicoccum* genus; *Bot*: *Botryosphaeriaceae* family.

**Table 3:** Comparison of real-time PCR (qPCR) sensitivities for the detection of *Botryosphaeriaceae* fungi in serial dilutions of DNA from an artificially inoculated almond twig extracted with a commercial kit (DNA extraction) or by direct sample preparation method (plant crude extracts).

Target Organism	Dilution	Real-Time PCR (Cq) ^1^	
		DNA Extraction	Plant Crude Extracts
*Neofusicoccum parvum*	1:1	+ (22.92 ± 0.10)	-
	1:10	+ (26.35 ± 0.04)	+ (29.24 ± 0.02)
	1:10^2^	+ (29.70 ± 0.07)	+ (31.52 ± 0.42)
	1:10^3^	+ (32.97 ± 0.09)	+ (35.18 ± 0.98)
	1:10^4^	+ (36.08 ± 0.43)	+ (38.88 ± 0.00)
	1:10^5^	+ (39.12 ± 0.00)	+/−
	1:10^6^	+/−	−
	1:10^7^	−	−
*Botryosphaeria dothidea*	1:1	+ (22.73 ± 0.07)	−
	1:10	+ (26.80 ± 0.13)	+ (32.71 ± 0.20)
	1:10^2^	+ (30.44 ± 0.11)	+ (35.90 ± 0.28)
	1:10^3^	+ (33.63 ± 0.14)	+ (38.84 ± 0.16)
	1:10^4^	+ (36.91 ± 0.29)	+ (42.85 ± 0.00)
	1:10^5^	+ (39.41 ± 0.45)	−
	1:10^6^	+/−	−
	1:10^7^	−	−

^1^ + Positive amplification; − negative amplification; +/− amplification in one or two out of the three replicates. Cq values (threshold cycle of the real-time PCR) ± standard error of three replicates is in parenthesis.

**Table 4:** Fungal isolates used in this study.

Species	Isolate ID	Host	Year of Isolation	Country
*Botryosphaeria dothidea*	Bd ALM1	*Prunus dulcis*	2016	Spain
	Bd ALM2	*Prunus dulcis*	2016	Spain
	Bd ALM3	*Prunus dulcis*	2016	Spain
	Bd ALM4	*Prunus dulcis*	2021	Spain
	Bd ALM6	*Prunus dulcis*	2021	Spain
	Bd ALM7	*Prunus dulcis*	2021	Spain
	Bd ALM8	*Prunus dulcis*	2021	Spain
	Bd ALM9	*Prunus dulcis*	2021	Spain
	Bd ALM10	*Prunus dulcis*	2022	Spain
	Bd ALM11	*Prunus dulcis*	2022	Spain
	Bd ALM12	*Prunus dulcis*	2022	Spain
	Bd ALM13	*Prunus dulcis*	2022	Spain
	Bd ALM14	*Prunus dulcis*	2022	Spain
	Bd ALM15	*Prunus dulcis*	2022	Spain
	Bd ALM16	*Prunus dulcis*	2022	Spain
	Bd ALM17	*Prunus dulcis*	2022	Spain
	ALM TOR1	*Prunus dulcis*	2022	Spain
	Bo.13.2	*Vaccinium corymbosum*	2009	Spain
	Bd1141	*Vitis vinifera*	-	Spain
	Bd1143	*Vitis vinifera*	-	Spain
*Botryosphaeria* sp.	Bo.11	*Vaccinium corymbosum*	2009	Spain
*Neofusicoccum australe*	Bo.8	*Vaccinium corymbosum*	2009	Spain
*Neofusicoccum luteum*	NF 146	*Persea americana*	2013	Spain
*Neofusicoccum mediterraneum*	Nm ALM3	*Prunus dulcis*	2020	Spain
	CJL 593	*Pistacia vera*	2005	Spain
*Neofusicoccum parvum*	Np ALM1	*Prunus dulcis*	2018	Spain
	Np ALM2	*Prunus dulcis*	2019	Spain
	Np ALM5	*Prunus dulcis*	2022	Spain
	NF 152	*Persea americana*	2013	Spain
	NF 161	*Persea americana*	2013	Spain
	Bo.2	*Vaccinium corymbosum*	2009	Spain
	Bo.4.1	*Vaccinium corymbosum*	2009	Spain
	Bo.4.2	*Vaccinium corymbosum*	2009	Spain
	Bo.6.1	*Vaccinium corymbosum*	2009	Spain
	Bo.7	*Vaccinium corymbosum*	2009	Spain
	Bo.9	*Vaccinium corymbosum*	2009	Spain
	Bo.10	*Vaccinium corymbosum*	2009	Spain
	Bo.13.3	*Vaccinium corymbosum*	2009	Spain
	Bo.14.2	*Vaccinium corymbosum*	2009	Spain
	Bo.16	*Vaccinium corymbosum*	2009	Spain
	Bo.17.1	*Vaccinium corymbosum*	2009	Spain
*Diplodia cortícola*	CJL 165	*Quercus suber*	1995	Spain
	CJL 166	*Quercus suber*	1995	Spain
*Diplodia cupresii*	GIHF 321	*Vitis vinifera*	2021	Spain
*Diplodia mutila*	CJL 456	*Fraxinus excelsior*	2003	Spain
*Diplodia seriata*	Ds ALM1	*Prunus dulcis*	2019	Spain
	CJL 398	*Vitis vinifera*	2003	Spain
*Dothiorella fraxini*	GIHF 132	*Fraxinus angustifolia*	2016	Spain
*Dothiorella iberica*	CJL 218	*Quercus ilex*	1999	Spain
	CJL 220	*Quercus ilex*	1999	Spain
*Dothiorella viticola*	CJL 570	*Vitis vinifera*	2004	Spain
	CJL 572	*Vitis vinifera*	2004	Spain
*Lasiodiplodia theobromae*	L.2	*Vaccinium corymbosum*	2017	Spain
	GIHF 272	*Vitis vinifera*	2019	Spain
*Macrophomina phaseolina*	Mp ALM 1	*Prunus dulcis*	2018	Spain
	Mp ALM 2	*Prunus dulcis*	2019	Portugal
	Mp ARA11	*Vaccinium corymbosum*	2018	Spain
	Mp ARA12	*Vaccinium corymbosum*	2019	Spain
	TOR 872	*Vaccinium corymbosum*	2017	Spain
	TOR 956	*Vaccinium corymbosum*	2020	Spain
*Cytospora acaciae*	Ca ALM1	*Prunus dulcis*	2022	Spain
	Ca ALM2	*Prunus dulcis*	2022	Spain
	Ca ALM3	*Prunus dulcis*	2022	Spain
*Botrytis cinerea*	Bc ARA1	*Vaccinium corymbosum*	2021	Spain
	Bc ALM1	*Prunus dulcis*	2016	Spain
*Monilia fructicola*	Mf CIR1	*Prunus salicina*	2011	Spain
*Monilia laxa*	Ml CIR1	*Prunus salicina*	2011	Spain
*Diaporthe amygdali*	DAL-65	*Prunus dulcis*	2017	Spain
*Diaporthe foeniculina*	DAL-69	*Prunus dulcis*	2017	Spain
*Diaporthe phaseolorum*	DAL-222	*Prunus dulcis*	2018	Spain
*Collectotrichum accutatum*	20,240	CECT	-	Spain
*Verticilium dahliae*	Vd ALM1	*Prunus dulcis*	2017	Spain
*Cylindrocladiella variabilis*	AL139	*Prunus dulcis*	2019	Spain
*Dactylonectria macrodidyma*	AL150	*Prunus dulcis*	2019	Spain
*Dactylonectria novozelandica*	AL84	*Prunus dulcis*	2019	Spain
*Dactylonectria torresensis*	AL3	*Prunus dulcis*	2019	Spain
*Ilyonectria liriodendri*	AL79	*Prunus dulcis*	2019	Spain
*Neonectria quercicola*	AL141	*Prunus dulcis*	2019	Spain
*Rhizoctonia solani*	Rs ALM4	*Prunus dulcis*	2020	Spain
*Epicoccum nigrum*	En ALM5	*Prunus dulcis*	2020	Spain
*Alternaria alternata*	Al ALM1	*Prunus dulcis*	2020	Spain

CECT: Colección Española de Cultivos Tipo (https://www.uv.es/uvweb/coleccion-espanola-cultivos-tipo/es/coleccion-espanola-cultivos-tipo-1285872233521.html (accessed on 6 February 2023).

## Data Availability

Not applicable.

## Supplementary Materials

The following supporting information can be downloaded at: https://www.mdpi.com/article/10.3390/plants12112205/s1, Table S1. GenBank Accession number of rRNA internal transcribed spacer (ITS) and translation elongation factor 1 alpha (*tef1*) gene of *Botryosphaeriaceae* isolates from almond, avocado and blueberry. Table S2. *In silico* analysis of the specificity of qPCR primers and probes for the detection of *Neofusicoccum parvum* and *Botryosphaeria dothidea* affecting Mediterranean woody crops. Reference [62] is cited in the supplementary materials.
