# Supplementary material for: Duplex Real-Time PCR Assays for the Simultaneous Detection and Quantification of Botryosphaeriaceae Species Causing Canker Diseases in Woody Crops
Source: Plants (Basel). 2023 Jun 2;12(11):2205. doi: 10.3390/plants12112205 (PMC10255876; doi:10.3390/plants12112205)
Supplement: Supplementary file 1 [file plants-12-02205-s001.zip › plants-2333406-supplementary.pdf]

Supplementary Table S1. GenBank Accession number of rRNA internal transcribed spacer (ITS) and translation elongation factor 1 alpha (*tef1*) gene of *Botryosphaeriaceae* isolates from almond, avocado and blueberry.

Species	Isolate ID	Host	Variety	GenBank Accession number	
				ITS	*tef1*
Botryosphaeria dothidea	Bd ALM1	Almond	-	OQ672339	OQ676462
	Bd ALM2	Almond	Marta	OQ672340	OQ676463
	Bd ALM3	Almond	Guara	OQ672341	OQ676464
	Bd ALM4	Almond	Soleta	OQ672342	OQ676465
	Bd ALM6	Almond	Soleta	OQ672343	OQ676466
	Bd ALM7	Almond	Vairo	OQ672344	OQ676467
	Bd ALM8	Almond	Vairo	OQ672345	OQ676468
	Bd ALM9	Almond	Marcona	OQ672346	OQ676469
	Bd ALM10	Almond	Soleta	OQ672347	OQ676470
	Bd ALM11	Almond	Lauranne	OQ672348	OQ676471
	Bd ALM12	Almond	Lauranne	OQ672349	OQ676472
	Bd ALM13	Almond	Soleta	OQ672350	OQ676473
	Bd ALM14	Almond	Belona	OQ672351	OQ676474
	Bd ALM15	Almond	Belona	OQ672352	OQ676475
	Bd ALM16	Almond	Belona	OQ672353	OQ676476
	Bd ALM17	Almond	Belona	OQ672354	OQ676477
	ALMTOR1	Almond	Marcona	OQ672355	OQ676478
	Bo.13.2	Blueberry	-	KC556959	KC556963
Neofusicoccum parvum	Np ALM1	Almond	Lauranne	OQ672356	OQ676479
	Np ALM2	Almond	Rootpac-40	OQ672357	OQ676480
	Np ALM5	Almond	Soleta	OQ672358	OQ676481
	NF 161	Avocado	Hass	OQ672359	OQ676482
	NF 152	Avocado	Hass	OQ672360	OQ676483
	Bo.6.1	Blueberry	-	KC556960	KC556961
Neofusicoccum luteum	NF 146	Avocado	Hass	OQ672362	OQ676485
Neofusicoccum mediterraneum	Nm ALM3	Almond	Rootpac-40	OQ672361	OQ676484
Neofusicoccum australe	Bo.8	Blueberry	-	KC556958	KC556962
Diplodia seriata	Ds ALM1	Almond	Guara	OQ672363	OQ676486
Macrophomina phaseolina	MpALM 1	Almond	GxN-15	OQ672364	OQ676487
Lasiodiplodia theobromae	L.2	Blueberry	-	MK542377	MK570085

Table S2. *In silico* analysis of the specificity of qPCR primers and probes for the detection of *Neofusicoccum parvum* and *Botryosphaeria dothidea* affecting Mediterranean woody crops

		Neofusicoccum parvum qPCR			*Botryosphaeria dothidea* qPCR			
		Forward primer	Reverse primer	Probe	Forward primer	Reverse primer	Probe	
**Host**	**Species detected in each crop**	**Np-F**	**Np-R**	**Np-P**	**Bd-F**	**Bd-R**	**Bd-P**	**References**
Almond	*Botryosphaeria dothidea*	-	-	-	+	+	+	16; 5; 13; 23; 18
	Diplodia corticola	-	-	-	+	-	-	15
	Diplodia gallae	-	-	-	-	-	-	18
	Diplodia mutila	-	-	-	-	-	-	16; 15; 18
	Diplodia olivarum	-	-	-	-	-	+	21; 5
	Diplodia seriata	-	-	-	-	-	+	21; 16; 5; 13; 15: 23; 18
	Neofusicoccum arbuti	+	+	-	-	-	-	16
	Neofusicoccum australe	-	-	-	-	-	-	5
	Neofusicoccum luteum	-	+	-	-	-	-	21
	Neofusicoccum mediterraneum	-	-	-	-	-	-	21; 16; 13; 15; 23
	Neofusicoccum nonquaesitum	+	+	-	-	-	-	13; 23
	Neofusicoccum parvum	+	+	+	-	-	-	21; 16; 5; 13; 15; 18
	Neofusicoccum vitifusiforme	+	-	-	-	-	-	16
	Dothiorella iberica	-	-	-	-	-	-	16; 15; 23
	Dothiorella prunícola	-	-	-	-	-	-	25
	Dothiorella sarmentorum	-	-	-	-	-	-	13; 15; 23
	Dothiorella viticola	-	-	-	-	-	-	16; 15
	Lasiodiplodia theobromae	-	-	-	-	-	-	16; 18; 22
	Neoscytalidium dimidiatum	-	-	-	-	-	-	16; 15; 23
	Macrophomina phaseolina	-	-	-	-	-	-	13; 23
	Botryosphaeria dothidea	-	-	-	+	+	+	46
	Diplodia cortícola	-	-	-	+	-	-	25
	Diplodia mutila	-	-	-	-	-	-	46
	Diplodia seriata	-	-	-	-	-	+	25
	Diplodia sp.1	-	-	-	-	-	-	25

Grapevine	*Dothiorella sarmentorum*	-	-	-	-	-	-	25
	Dothiorella striata	-	-	-	-	-	-	25
	Dothiorella vinea-gemmae	-	-	-	-	-	-	25
	Dothiorella viticola	-	-	-	-	-	-	46
	Lasiodiplodia crassispora	-	-	-	-	-	-	25
	Lasiodiplodia exigua	-	-	-	-	-	-	46
	Lasiodiplodia gilanensis	-	-	-	-	-	-	25
	Lasiodiplodia mahajangana	-	-	-	-	-	-	25
	Lasiodiplodia mediterranea	-	-	-	-	-	-	25
	Lasiodiplodia missouriana	-	-	-	-	-	-	46
	Lasiodiplodia plurivora	-	-	-	-	-	-	25
	Lasiodiplodia theobromae	-	-	-	-	-	-	46
	Lasiodiplodia viticola	-	-	-	-	-	-	25
	Lasiodiplodia vitis	-	-	-	-	-	-	25
	Neofusicoccum australe	-	-	-	-	-	-	46
	Neofusicoccum kwambonambiense	+	+	-	-	-	-	48
	Neofusicoccum luteum	-	+	-	-	-	-	48
	Neofusicoccum mediterraneum	-	-	-	-	-	-	46
	Neofusicoccum parvum	+	+	+	-	-	-	46
	Neofusicoccum ribis	+	+	+	-	-	-	52
	Neofusicoccum stellenboschiana	-	+	-	-	-	-	25
	Neofusicoccum viticlavatum	+	-	-	-	-	-	25
	Neofusococcum vitifusiforme	+	-	-	-	-	-	25
	Neoscytalidium dimidiatum	-	-	-	-	-	-	46
	Neoscytalidium sp.1	-	-	-	-	-	-	25
	Sphaeropsis porosa	-	-	-	-	-	-	25
	Botryosphaeria dothidea	-	-	-	+	+	+	5
	Diplodia africana	-	-	-	-	-	-	5
	Diplodia mutila	-	-	-	-	-	-	5
	Diplodia pinea	-	-	-	-	-	-	5
	Diplodia rosulata	-	-	-	-	-	-	5
	Diplodia sapinea	-	-	-	-	-	-	25

Prunus spp.	*Diplodia seriata*	-	-	-	-	-	+	5
	Dothiorella iberica	-	-	-	-	-	-	46
	Dothiorella sarmentorum	-	-	-	-	-	-	46
	Lasiodiplodia plurívora	-	-	-	-	-	-	5
	Lasiodiplodia pseudotheobromae	-	-	-	-	-	-	51
	Lasiodiplodia thebromae	-	-	-	-	-	-	5
	Macrophomina phaseolina	-	-	-	-	-	-	5
	Neofusicoccum australe	-	-	-	-	-	-	5
	Neofusicoccum mediterraneum	-	-	-	-	-	-	5
	Neofusicoccum nonquaesitum	+	+	-	-	-	-	5
	Neofusicoccum parvum	+	+	+	-	-	-	5
	Neofusicoccum ribis	+	+	+	-	-	-	5
	Neofusicoccum stellenboschiana	-	+	-	-	-	-	25
	Neofusicoccum vitifusiforme	+	-	-	-	-	-	5
	Neoscytalidium dimidiatum	-	-	-	-	-	-	51
	Spencermartinsia viticola	-	-	-	-	-	-	5
Walnut	*Botryosphaeria dothidea*	-	-	-	+	+	+	24
	Diplodia mutila	-	-	-	-	-	-	24
	Diplodia seriata	-	-	-	-	-	+	24
	Dothiorella iberica	-	-	-	-	-	-	24
	Lasiodiplodia citricola	-	-	-	-	-	-	46
	Lasiodiplodia gilanensis	-	-	-	-	-	-	24
	Lasiodiplodia theobromae	-	-	-	-	-	-	24
	Neofusicoccum luteum	-	+	-	-	-	-	24
	Neofusicoccum mediterraneum	-	-	-	-	-	-	24
	Neofusicoccum nonquasitum	+	+	-	-	-	-	46; 24
	Neofusicoccum parvum	+	+	+	-	-	-	24
	Neofusicoccum vitifusiforme	+	-	-	-	-	-	46
	Neoscytalidium dimidiatum	-	-	-	-	-	-	24
	Botryosphaeria dothidea	-	-	-	+	+	+	24
	Diplodia pseudoseriata	-	-	-	-	-	-	25

Pistachio	*Diplodia seriata*	-	-	-	-	-	+	24
	Dothiorella iberica	-	-	-	-	-	-	24
	Dothiorella sarmentorum	-	-	-	-	-	-	24
	Dothiotella viticola	-	-	-	-	-	-	24
	Lasiodiplodia americana	-	-	-	-	-	-	24
	Lasiodiplodia citricola	-	-	-	-	-	-	24
	Lasiodiplodia gilanensis	-	-	-	-	-	-	24
	Lasiodiplodia pseudotheobromae	-	-	-	-	-	-	51
	Lasiodiplodia theobromae	-	-	-	-	-	-	24
	Macrophomina phaseolina	-	-	-	-	-	-	24
	Neofusicoccum australe	-	-	-	-	-	-	24
	Neofusicoccum hellenicum	+	-	-	-	-	-	46
	Neofusicoccum mediterraneum	-	-	-	-	-	-	24
	Neofusicoccum parvum	-	-	-	-	-	-	24
	Neofusicoccum pistaciae	-	-	-	-	-	-	25
	Neofusicoccum vitifusiforme	+	-	-	-	-	-	24
	Neoscytalidium dimidiatum	-	-	-	-	-	-	24
Olive	*Barriopsis barriana*	-	-	-	-	-	-	25
	Botryosphaeria dothidea	-	-	-	+	+	+	24
	Diplodia cortícola	-	-	-	+	-	-	24
	Diplodia mutila	-	-	-	-	-	-	24
	Diplodia olivarum	-	-	-	-	-	-	24
	Diplodia pinea	-	-	-	-	-	-	24
	Diplodia scrobiculata	-	-	-	-	-	-	24
	Diplodia seriata	-	-	-	-	-	+	24
	Dothiorella iberica	-	-	-	-	-	-	24
	Dothiorella iranica	-	-	-	-	-	-	25
	Lasiodiplodia hormozganensis	-	-	-	-	-	-	25
	Lasiodiplodia pseudotheobromae	-	-	-	-	-	-	51
	Lasiodiplodia theobromae	-	-	-	-	-	-	24
	Macrophomina phaseolina	-	-	-	-	-	-	24
	Neofusicoccum australe	-	-	-	-	-	-	24

	Neofusicoccum luteum	-	+	-	-	-	-	24
	Neofusicoccum mediterraneum	-	-	-	-	-	-	24
	Neofusicoccum parvum	+	+	+	-	-	-	24
	Neofusicoccum vitifusiforme	+	-	-	-	-	-	24
	Botryosphaeria dothidea	-	-	-	+	+	+	12
	Diplodia mutila	-	-	-	-	-	-	12
	Diplodia pseudoseriata	-	-	-	-	-	-	12
	Diplodia seriata	-	-	-	-	-	+	12
	Dothiorella iberica	-	-	-	-	-	-	12
	Lasiodiplodia mahajangana	-	-	-	-	-	-	25
	Lasiodiplodia pseudothebromae	-	-	-	-	-	-	12
	Lasiodiplodia thebromae	-	-	-	-	-	-	12
Avocado	*Neofusicoccum australe*	-	-	-	-	-	-	12
	Neofusicoccum luteum	-	+	-	-	-	-	12
	Neofusicoccum mangiferae	+	+	-	-	-	-	12
	Neofusicoccum mediterraneum	-	-	-	-	-	-	12
	Neofusicoccum nonquasitum	+	+	-	-	-	-	12
	Neofusicoccum parvum	+	+	+	-	-	-	12
	Neofusicoccum ribis	+	+	+	-	-	-	25
	Neofusicoccum stellenboschiana	-	+	-	-	-	-	25
	Pseudofusicoccum kimberleyense	-	-	-	-	-	-	25
	Barriopsis stevensiana	-	-	-	-	-	-	25
	Botryosphaeria fabicerciana	-	-	-	+	-	-	49
	Diplodia citricarpa	-	-	-	-	-	-	25
	Diplodia mutila	-	-	-	-	-	-	62
	Diplodia seriata	-	-	-	-	-	+	62
	Dothiorella citrícola	-	-	-	-	-	-	25
	Dothiorella iberica	-	-	-	-	-	-	62
	Dothiorella plurívora	-	-	-	-	-	-	25
	Dothiorella striata	-	-	-	-	-	-	25
	Dothiorella vitícola	-	-	-	-	-	-	62

Citrus spp.	*Lasiodiplodia citricola*	-	-	-	-	-	-	62
	Lasiodiplodia gilanensis	-	-	-	-	-	-	25
	Lasiodiplodia hormozganensis	-	-	-	-	-	-	62
	Lasiodiplodia iraniensis	-	-	-	-	-	-	62
	Lasiodiplodia mahajangana	-	-	-	-	-	-	25
	Lasiodiplodia parva	-	-	-	-	-	-	62
	Lasiodiplodia pseudothebroame	-	-	-	-	-	-	62
	Lasiodiplodia theobromae	-	-	-	-	-	-	62
	Lasiodiplodia subglosa	-	-	-	-	-	-	62
	Neofusicoccum australe	-	-	-	-	-	-	62
	Neofusicoccum mediterraneum	-	-	-	-	-	-	62
	Neofusicoccum parvum	+	+	+	-	-	-	62
	Neoscytalidium dimidiatum	-	-	-	-	-	-	62
	Neoscytalidium hyalinum	-	-	-	-	-	-	62
	Spencermartinsia viticola	-	-	-	-	-	-	62
	Sphaeropsis citrigena	-	-	-	-	-	-	25
Blueberry	*Botryosphaeria dothidea*	-	-	-	+	+	+	48; 25
	Botryosphaeria corticis	-	-	-	+	-	-	48
	Lasiodiplodia citrícola	-	-	-	-	-	-	25
	Lasiodiplodia laeliocattleyae	-	-	-	-	-	-	53
	Lasiodiplodia lignícola	-	-	-	-	-	-	25
	Lasiodiplodia pesudothebromae	-	-	-	-	-	-	53
	Lasiodiplodia thebromae	-	-	-	-	-	-	53
	Neofusicoccum arbuti	+	+	-	-	-	-	53
	Neofusicoccum australe	-	-	-	-	-	-	48
	Neofusicoccum eucaliptorum	-	+	-	-	-	-	48
	Neofusicoccum nonquaesitum	+	+	-	-	-	-	25
	Neofusicoccum kwambonambiense	+	+	-	-	-	-	53
	Neofusicoccum parvum	+	+	+	-	-	-	48
	Neofusicoccum ribis	+	+	+	-	-	-	53

62. Mondragón-Flores A, Rodríguez-Alvarado G, Gómez-Dorantes N, Guerra-Santos JJ, Fernández-Pavía SP (2021). *Botryosphaeriaceae* : a complex, diverse and cosmopolitan family of fungi. Revista Mexicana Ciencias Agrícolas volume 12 number 4 May 16
